# Expanding the Patient Pool for Rilonacept

**DOI:** 10.1016/j.jaccas.2025.103799

**Published:** 2025-07-03

**Authors:** Emma St. John, Jessica R. Carey, Tanya Wilcox, John Ryan, Satvik Ramakrishna, Anees Daud, Denise Diaz-Robles, Adam Smith, Kristin Cowan, Libo Wang

**Affiliations:** University of Utah Health, Salt Lake City, Utah, USA

**Keywords:** interleukin-1, recurrent pericarditis, rilonacept

## Abstract

Recurrent pericarditis complicates 15%-30% of acute pericarditis cases despite initial anti-inflammatory treatment. The RHAPSODY trial (Study to Assess the Efficacy and Safety of Rilonacept Treatment in Participants With Recurrent Pericarditis) showed rilonacept, an interleukin-1 cytokine trap, to be an effective therapeutic option for patients with pericarditis recurrence. This case series demonstrates the utility and challenges of rilonacept use in the types of patients excluded from RHAPSODY.

## Graphical Abstract

**Figure:**
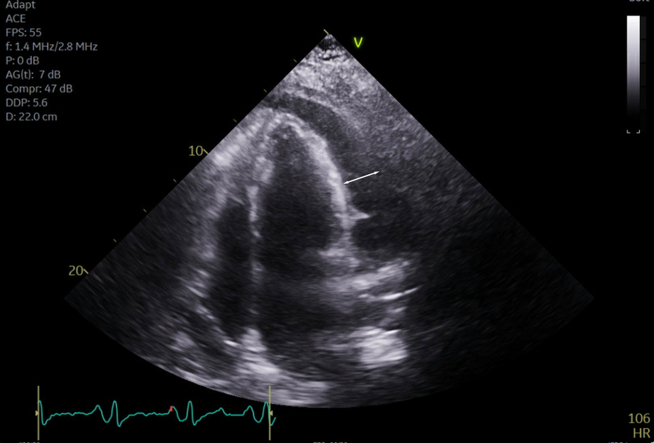

Recurrent pericarditis complicates 15%-30% of acute pericarditis cases despite initial anti-inflammatory treatment.^1^ After 2 or more episodes of recurrent pericarditis, up to 50% of patients will continue to experience recurrent episodes, often requiring long-term treatment.^2^ Rilonacept, an interleukin (IL)-1 cytokine trap, provides a therapeutic option for patients with continued pericarditis recurrence despite conventional therapies, as was demonstrated in RHAPSODY (Study to Assess the Efficacy and Safety of Rilonacept Treatment in Participants With Recurrent Pericarditis). The pathogenesis of pericarditis is presumed to be autoinflammatory or immune-mediated, with most cases being idiopathic. The RHAPSODY trial excluded patients with radiation-induced pericarditis, a history of systemic autoimmune disease, and posttraumatic pericarditis, and the trial had specific criteria for tapering anti-inflammatory agents during the run-in period.^3^ Our case series demonstrates the utility and challenges of prescribing rilonacept in patients outside of the inclusion and exclusion parameters of RHAPSODY.

**Take-Home Messages**

• Recurrent pericarditis is a complex disease that significantly effects the lives of patients and imposes a weighty demand on health care resources.
• Rilonacept, through the RHAPSODY trial, was demonstrated to be an effective therapeutic option for many individuals with recurrent pericarditis, but several important patient groups were excluded.
• Our cases demonstrate the challenges and lack of clear guidance when determining rilonacept use, use duration, and continuation parameters in cases of recurrent pericarditis in groups who would have been excluded from the RHAPSODY trial.
• Increasing use of cardiac MRI has been instrumental in the diagnosis and management of pericarditis, but limitations still exist in multimodal imaging in pericardial disease, especially in those with prior cardiac surgery or cardiectomy.

## Case 1

A 48-year-old woman was initially diagnosed with pericarditis in October 2023 after presenting with 2 days of chest pain. Three days before her initial presentation she had completed her last session of radiation for invasive ductal carcinoma. The diagnosis of pericarditis was made in the emergency department after a bedside echocardiogram demonstrated a small pericardial effusion in combination with elevated inflammatory markers. She was initially treated with a 10-day course of ibuprofen with symptom improvement but subsequent recurrence.

**Figure 1:**
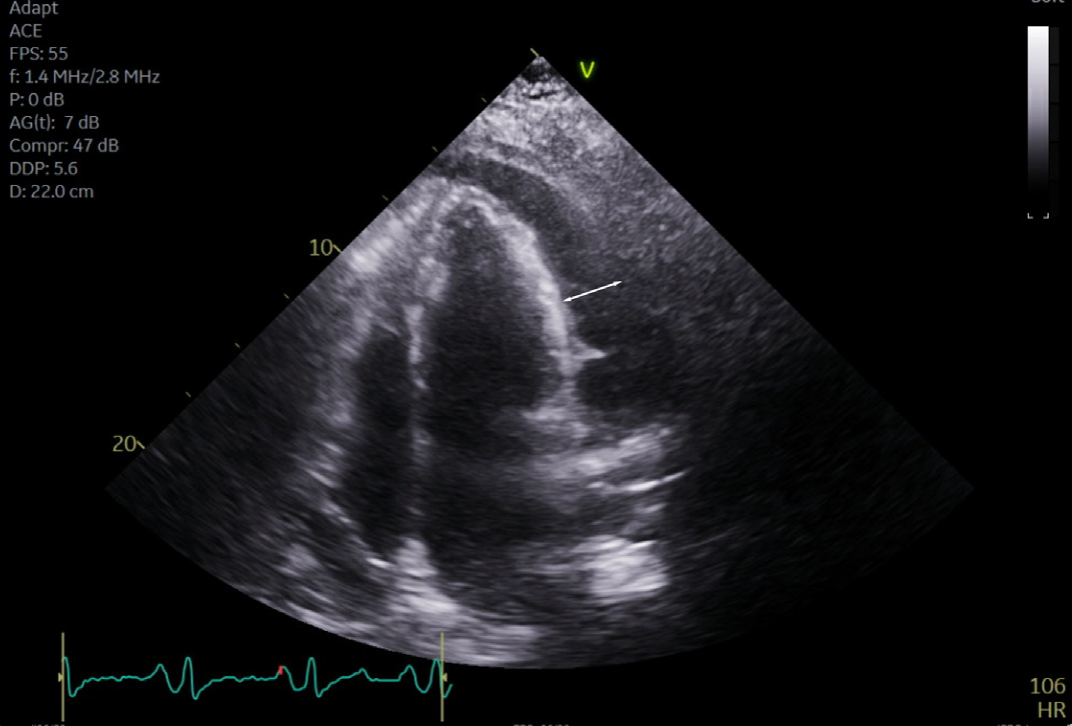
Transthoracic Echocardiogram Transthoracic echocardiogram demonstrating a large, circumferential pericardial effusion (white arrow) with significant features of cardiac tamponade, constriction, and focal strands.

**Figure 2:**
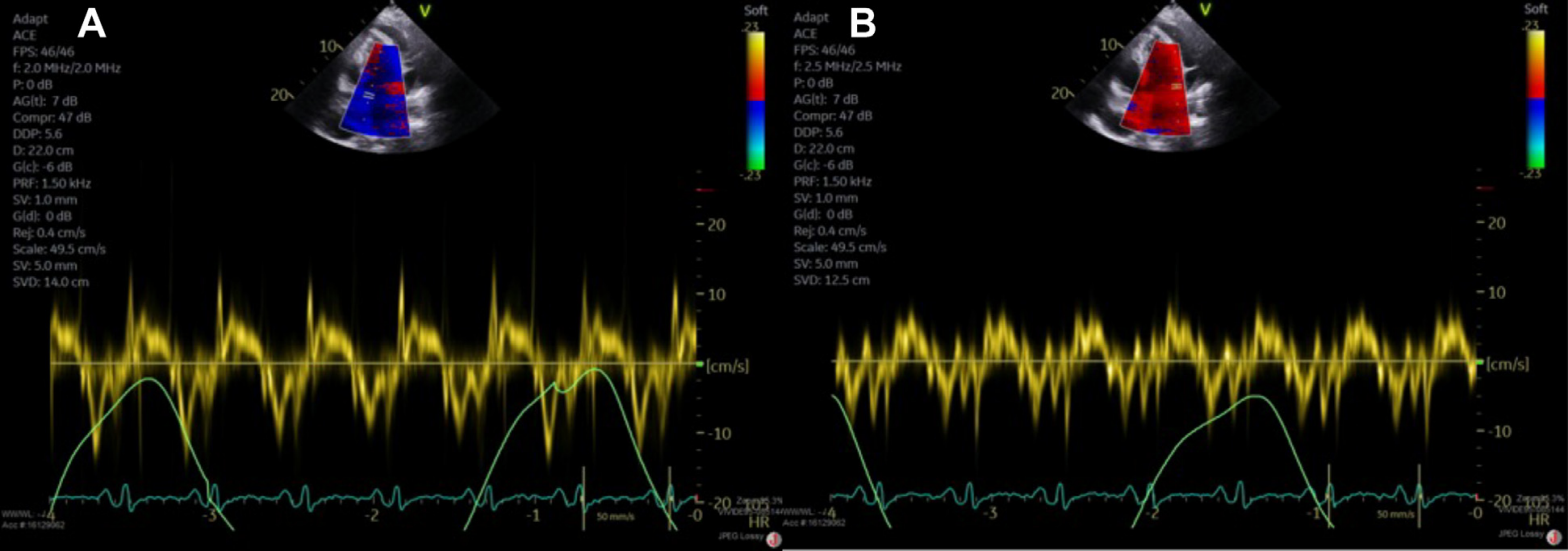
Transthoracic Echocardiogram With Tissue Doppler Echocardiogram demonstrating elevated medial mitral annular eʹ velocities greater than the lateral annular eʹ velocities, consistent with annulus reversus, and early tethering of the lateral mitral annulus with acute constrictive pericarditis.

She was seen in the emergency department 3 more times and was prescribed short courses of nonsteroidal anti-inflammatory drugs (NSAIDs). She was started on colchicine and a prolonged course of ibuprofen in January 2024 with continued chest pain. Eventually, she was admitted to the hospital for worsening dyspnea and was found to have a large pericardial effusion with features of effusive constrictive pericarditis with mitral annulus reversus (Figures 1 and 2).

**Figure 3:**
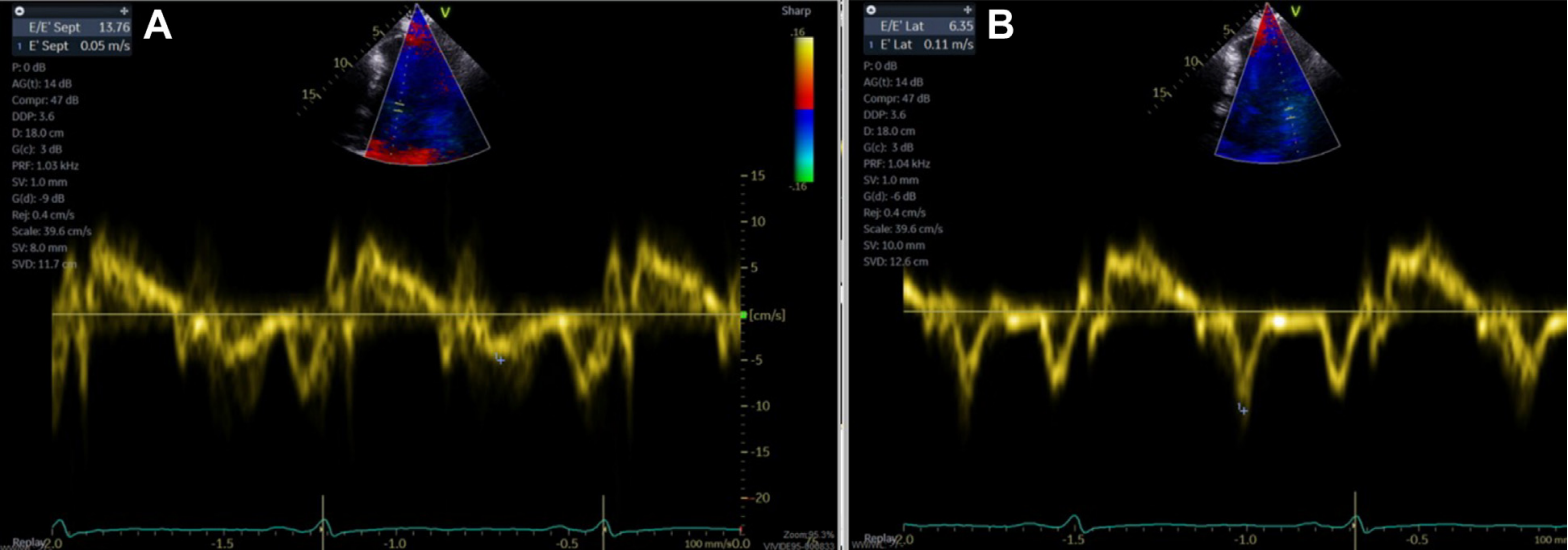
Transthoracic Echocardiogram With Tissue Doppler After Immunosuppressive Therapy Echocardiogram demonstrating normalization of the relationship between medial eʹ and lateral eʹ velocities after immunosuppressive treatment.

Pericardiectomy was eventually performed with resolution of the annulus reversus (Figure 3). Colchicine and high-dose NSAIDs did not improve her symptoms or the inflammatory markers after the procedure. Prednisone was initiated at 60 mg/day, and a taper of 10 mg/week was tolerated until 20 mg daily before chest pain recurrence and C-reactive protein (CRP) increase.

Rilonacept was initiated April 2024 due to her continued chest pain despite prednisone, colchicine, and NSAIDs with the plan to taper the prednisone by 5 mg/week with discontinuation of colchicine and ibuprofen. She was able to tolerate the discontinuation of colchicine and ibuprofen and a prednisone taper to 15 mg/day with the addition of rilonacept. After 2 months of rilonacept, she was able to discontinue steroids and proceed on rilonacept monotherapy.

**Figure 4:**
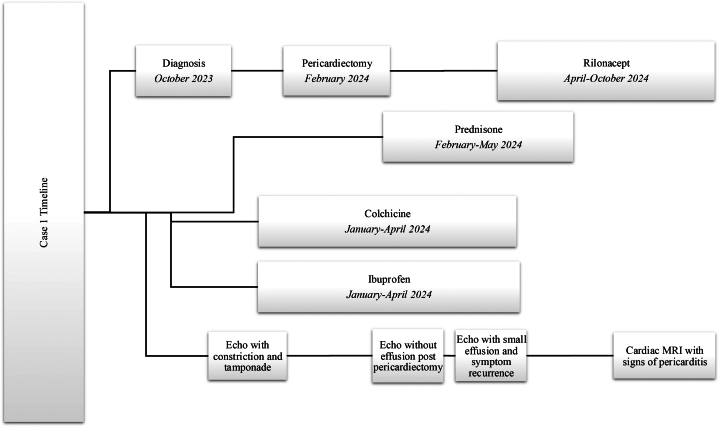
Timeline of Events, Case 1 General timeline of diagnosis, therapies including rilonacept and benchmark imaging. Echo = echocardiogram; MRI = magnetic resonance imaging.

Four months into monotherapy, her symptoms have recurred. Cardiac magnetic resonance imaging (MRI) demonstrated findings compatible with pericarditis. Discussions are ongoing about restarting the adjuvant therapy (Figure 4).

## Case 2

A 37-year-old woman with a history of fibromyalgia and polycystic ovary syndrome, requiring monthly triamcinolone injections from 2015-2024, initially presented to the emergency department in February 2022 with body aches and chest pain. She was sent home without treatment. She returned to the emergency department twice more and was again sent home without treatment.

At the end of March 2022, she presented to the emergency department with chest pain, fever, and leukocytosis as well as a small pericardial effusion and pericardial thickening. She completed two 2-week courses of high-dose naproxen and did not tolerate greater than a 2-week course of colchicine. Despite therapy, she continued to have recurrent chest pain.

**Figure 5:**
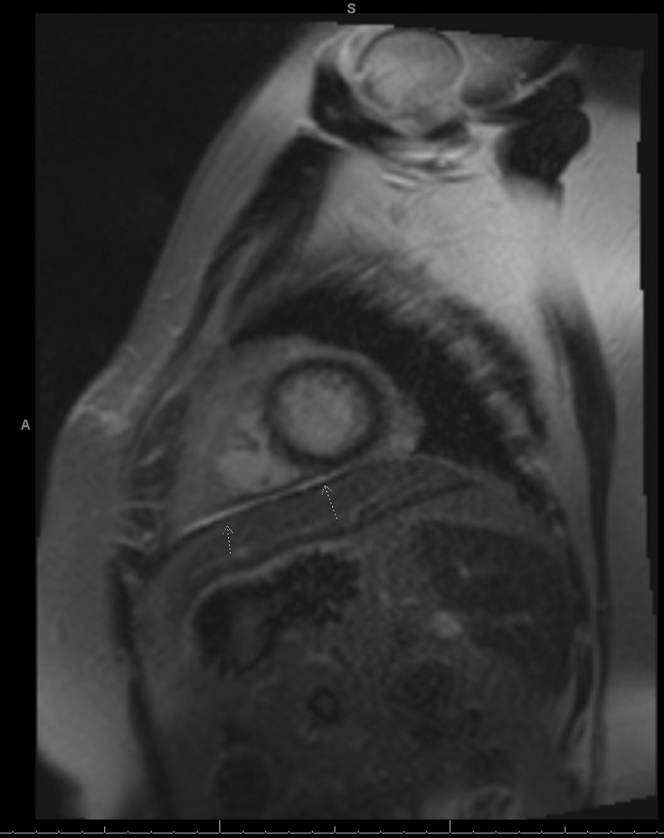
Cardiac Magnetic Resonance Imaging Cardiac magnetic resonance with thickened pericardium in the inferior aspect of the heart (arrow), with abnormal late gadolinium enhancement uptake.

A cardiac MRI in October 2022 had findings consistent with pericarditis, with extensive delayed gadolinium enhancement of the pericardium (Figure 5). These findings were compared with a chest CT from June 2022 that showed findings compatible with calcification, suggestive of components of chronic pericarditis. Persistent Kussmaul sign was noted, with the cardiac MRI supportive of constrictive physiology. She was started on rilonacept in December 2022 with a continuation of concurrent high-dose aspirin and colchicine for the first 2 months. She had improvement in her symptoms, and her erythrocyte sedimentation rate, CRP, and white blood cell count were within normal limits before pericardiectomy.

**Figure 6:**
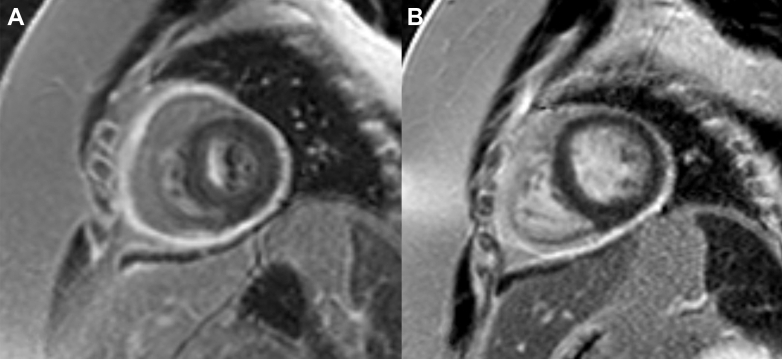
Cardiac Magnetic Resonance Imaging (A) Cardiac magnetic resonance (CMR) phase sensitive inversion recovery (PSIR) late gadolinium enhancement (LGE) sequence demonstrating increased thickness of the pericardium, and severe circumferential pericardial enhancement and increased thickness. (B) PSIR LGE sequence obtained after pericardiectomy, demonstrating reduced burden of residual patchy LGE in the inferior pericardium.

**Figure 7:**
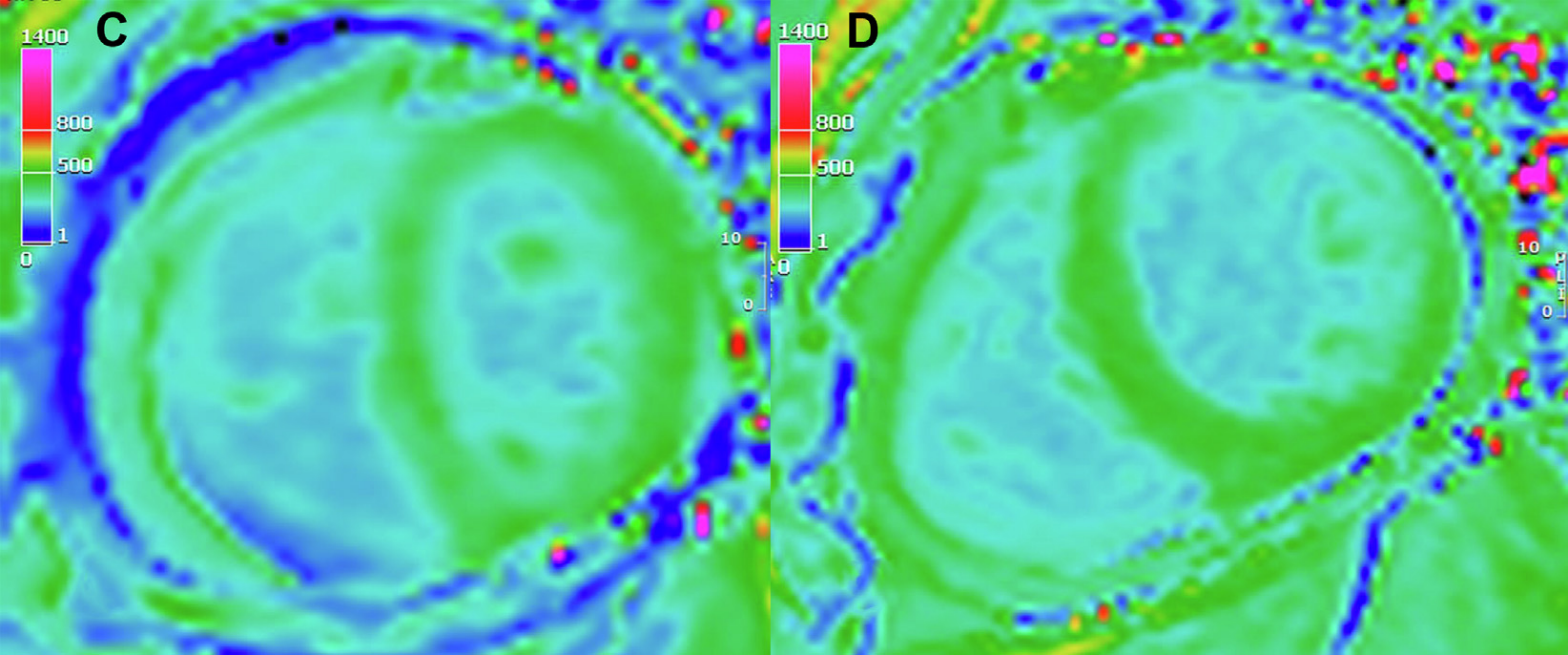
Cardiovascular Magnetic Resonance Imaging T1 Postcontrast Map (C) Corresponding T1 postcontrast map demonstrating short T1 time (dark blue) of the pericardium, consistent with pericarditis. (D) T1 postcontrast map after pericardiectomy with normalization of T1 changes.

**Figure 8:**
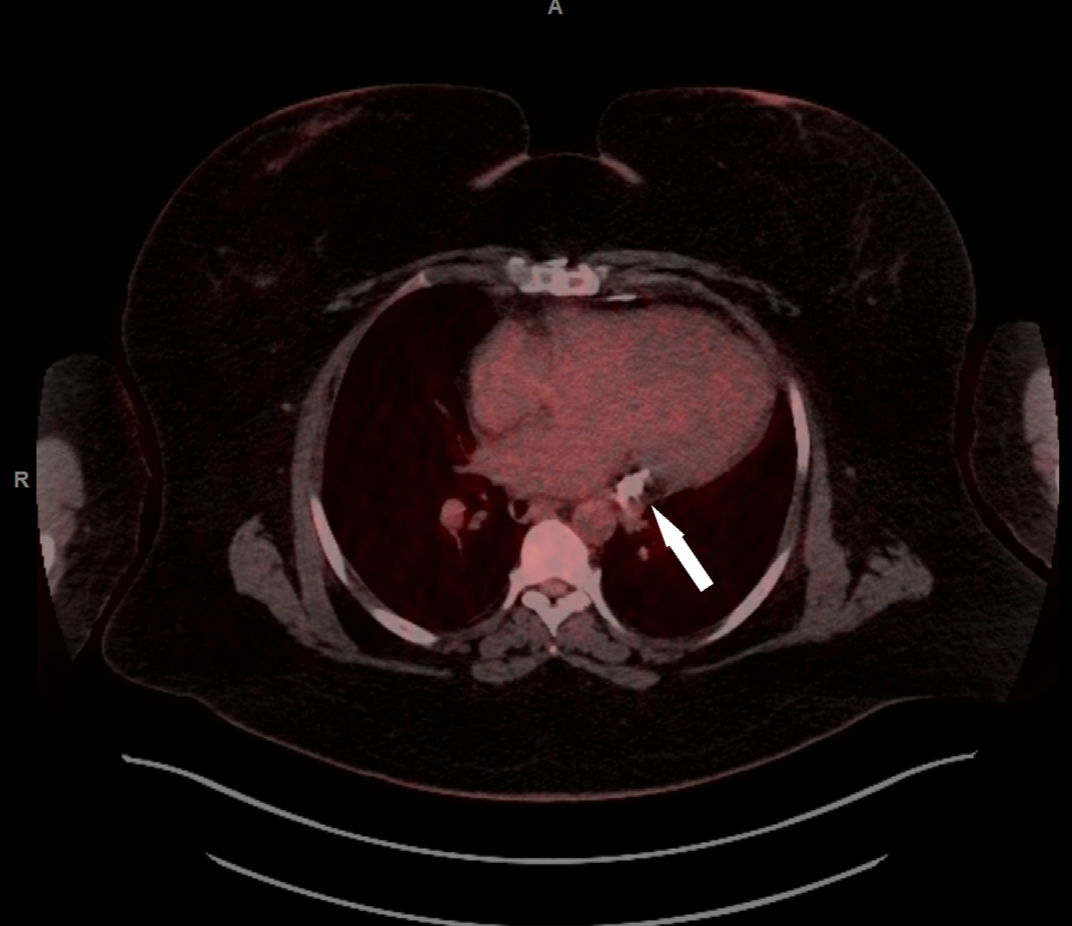
Fluorodeoxyglucose-Positron Emission Tomography Pericardiectomy fluorodeoxyglucose-positron emission tomography imaging of the heart. Imaging performed after 48 hours of a glucose-free/ketogenic diet. The hyperdensity (white arrow) demonstrating residual thickened and calcified pericardium near the base of the left ventricle. Note the absence of abnormal uptake within the myocardium or pericardial space.

A planned pericardiectomy was performed March 2023. Rilonacept was held before the pericardiectomy due to infection risk. Rilonacept was restarted after the procedure with recurrence of her chest pain, leading to the addition of a 70-day prednisone taper. An MRI in May 2024 showed a thickened posterior pericardium with pericardial delayed gadolinium enhancement without significant pericardial effusion (Figure 6). This is also seen in the corresponding T1 postcontrast map (Figure 7). The T2 mapping was poor quality and could not be used. A positron emission tomography (PET) scan at that time demonstrated no myocardial uptake that would suggest active myocardial inflammation (Figure 8), though the diagnostic performance of fluorodeoxyglucose (FDG) PET for idiopathic pericarditis is not well-elucidated and is likely less sensitive.

There was concern that, given these findings, it was hard to differentiate true inflammation from prior thickening with gadolinium uptake from scarred pericarditis or postsurgical residual pericardium with fibrosis. Given the possible risks of continued immunosuppression without a clear benefit, as demonstrated by her continued symptoms, and the lack of clear imaging findings, rilonacept was discontinued July 2024.

**Figure 9:**
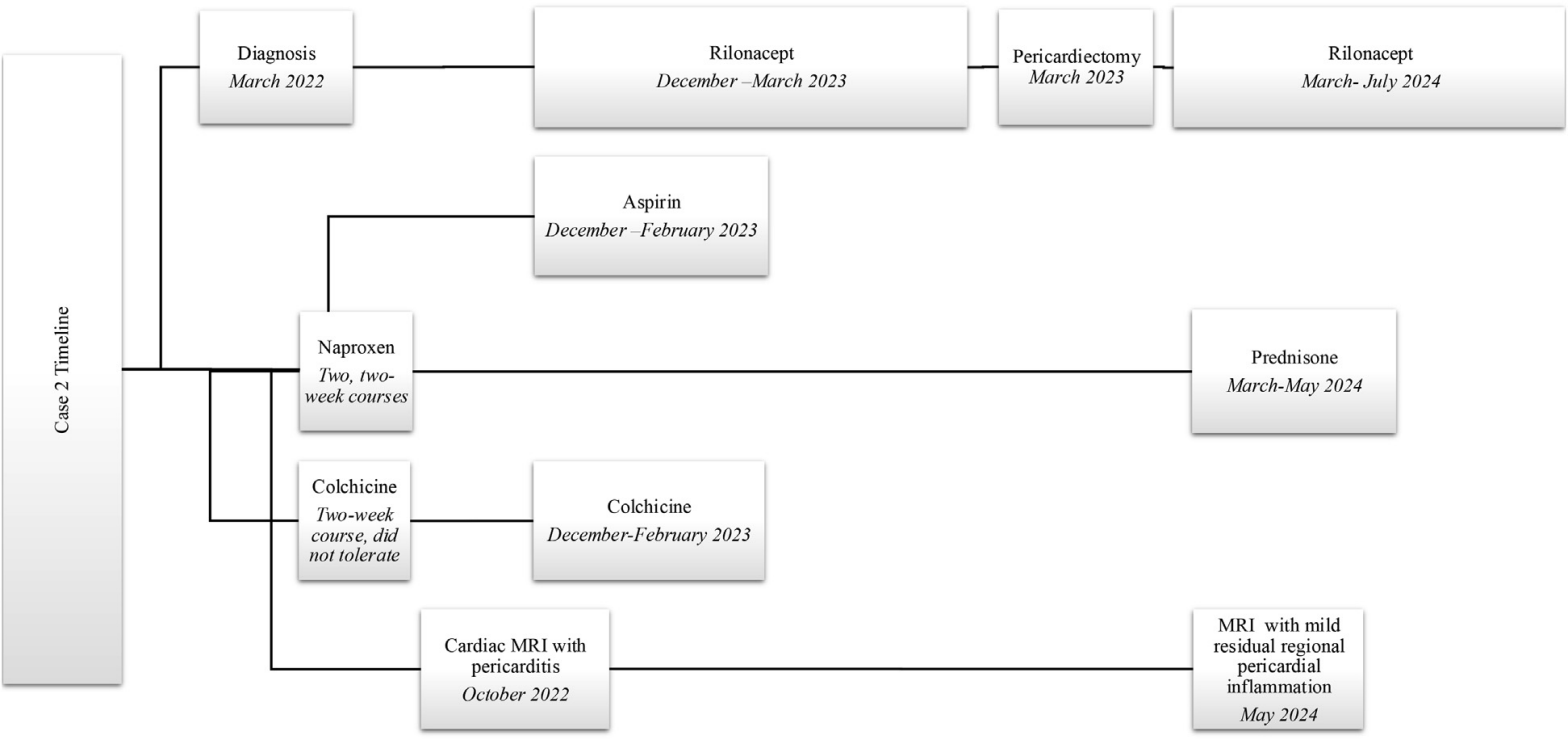
Timeline of Events, Case 2 General timeline of diagnosis, therapies including rilonacept, and benchmark imaging. MRI = magnetic resonance imaging.

After rilonacept discontinuation, the patient described improvement of her symptoms, and her inflammatory markers continued to be normal (Figure 9).

## Case 3

A 21-year-old man first presented to the emergency department in 2022 with chest pain and was subsequently discharged with a diagnosis of costochondritis. He presented 3 additional times with recurrent chest pain and received the diagnosis of costochondritis. Short courses of naproxen improved his symptoms.

His symptoms never completely resolved and worsened again in January 2024. He was prescribed a weeklong course of tramadol and prednisone with a suspected diagnosis of pericarditis, given his elevated inflammatory markers and a CT scan consistent with the diagnosis. An echocardiographic study revealed a pericardial effusion with fibrinous material. He subsequently completed two 2-week courses of naproxen with continued colchicine and one 15-day prednisone taper without improvement of symptoms.

During this time, the patient was referred to rheumatology. His laboratory results were notable for antinuclear antibodies of 1:160 in a speckled pattern with an elevated rheumatoid factor of 17 and persistently elevated erythrocyte sedimentation rate of 36. The double-stranded DNA (dsDNA), extractable nuclear antigen, and systemic sclerosis panels were negative. He was subsequently started on hydroxychloroquine in February 2024, in addition to the continued prednisone and colchicine. He was started on rilonacept in March 2024, given his continued chest pain; the adjuvant therapies were tapered until the patient was on rilonacept monotherapy.

**Figure 10:**
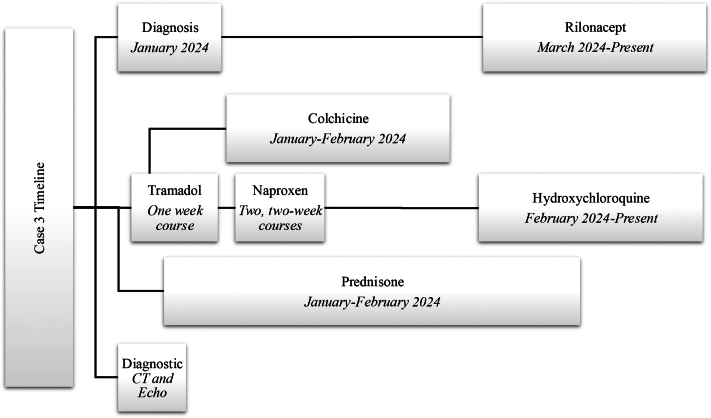
Timeline of Events, Case 3 General timeline of diagnosis, therapies including rilonacept, and benchmark imaging. CT = computed tomography; echo = echocardiogram.

Before rilonacept initiation, the patient’s CRP was elevated to 37.8 mg/dL, which decreased to 0.3 mg/dL within a month of starting rilonacept followed by a further decrease to <0.1 mg/dL. The patient reported complete resolution of his symptoms after the initiation of rilonacept. He continues on rilonacept and hydroxychloroquine without side effects and plans to dose reduce the hydroxychloroquine based on continued symptom resolution (Figure 10).

## Discussion

At a large academic center with expertise, and comfort in, prescribing rilonacept, we present 3 unique recurrent pericarditis cases that would have been excluded from the RHAPSODY trial due to a history of radiation, systemic immunosuppression, or autoimmune disease, respectively. Despite these exclusion criteria, we hypothesized that it is biologically plausibility that the IL-1 trap mechanism of rilonacept could extend to patients with inflammatory comorbidities because other IL-1 biologic agents have shown efficacy in treating a variety of inflammatory diseases outside of pericarditis. However, in these unique patient cases the rilonacept therapy had varied outcomes. Case 1 demonstrated initial but not maintained therapeutic success; case 2 illustrated a lack of clear improvement followed by rilonacept discontinuation; and Case 3 demonstrated therapeutic success.^4^^,^^5^ These cases highlight the uncertainty of rilonacept use in actual practice.

Case 1 demonstrated a lack of efficacy in patients with radiation-induced recurrent pericarditis, so further guidance is needed on best practices for nonresponders. Although this patient was able to achieve monotherapy with rilonacept, the symptoms recurred. In the RHAPSODY long-term extension trial, continued rilonacept treatment after 18 months led to continued results, with suspension of the treatment leading to recurrence of chest pain.^6^ It is possible that continued treatment in this case would have provided benefit, but questions still arise around the utility of rilonacept if a patient cannot remain on monotherapy and requires continued adjuvant therapy for symptom relief. If monotherapy continues to manage symptoms in patients requiring prolonged use, the question remains of when to discontinue therapy.

In case 2, rilonacept did not provide symptomatic relief after pericardiectomy. Diagnostic uncertainty was complicated by persistent late gadolinium enhancement on cardiac MRI contradicted by a lack of metabolic activity on FDG-PET and persistent chest pain with normal inflammatory markers.

By contrast with cases 1 and 2, case 3 demonstrated results similar to those of the RHAPSODY trial. The patient in case 3 saw complete resolution of symptoms and could taper all the adjuvant therapies after the initiation of rilonacept in concurrence with hydroxychloroquine. Although the diagnostic thresholds of cardiac MRI are well-elucidated in pericarditis in a patient after surgical cardiectomy, the clinical significance of pericardial late gadolinium enhancement in the post–cardiac surgery population has been called into question; its presence may signify fibrosis rather than inflammation.^7^ FDG-PET is used during the workup of neoplastic or infectious pericardial and systemic diseases, but specific diagnostic thresholds have not been validated in idiopathic pericarditis.

Despite the RHAPSODY long-term extension trial, there are still questions around when and how to taper rilonacept and its overall utility if the patient cannot otherwise tolerate adjuvant tapering with continued symptoms. Questions also arise around whether rilonacept should be discontinued in patients with persistent chest pain. Should there be a presumed lack of efficacy, or should rilonacept be continued with the hope of eventual resolution of pain—and if so, for how long? Continued data on the effectiveness and logistics of use of rilonacept in unique populations with recurrent pericarditis will allow for a better understanding of not only when but how to utilize rilonacept.

## Funding Support and Author Disclosures

Dr Ryan has served as a consultant for Kiniksa and has received grants from Kiniksa. All other authors have reported that they have no relationships relevant to the contents of this paper to disclose.
